# Sprint Interval Exercise Improves Cognitive Performance Unrelated to Postprandial Glucose Fluctuations at Different Levels of Normobaric Hypoxia

**DOI:** 10.3390/jcm11113159

**Published:** 2022-06-02

**Authors:** On-Kei Lei, Shengyan Sun, Jinlei Nie, Qingde Shi, Zhaowei Kong

**Affiliations:** 1 Faculty of Education, University of Macau, Macao, China; yb97106@um.edu.mo; 2 Institute of Physical Education, Huzhou University, Huzhou 313000, China; antun0605@163.com; 3 Faculty of Health Sciences and Sports, Macao Polytechnic University, Macao, China; jnie@ipm.edu.mo (J.N.); qdshi@ipm.edu.mo (Q.S.)

**Keywords:** high-intensity interval training, normoxia, executive function, glucose

## Abstract

Objective: The aim of our study was to examine cognition response to sprint interval exercise (SIE) against different levels of hypoxia. Research design and methods: 26 recreational active males performed SIE (20 × 6 s of all-out cycling bouts, 15 s of passive recovery) under normoxia (F_I_O_2_: 0.209), moderate hypoxia (F_I_O_2_: 0.154), and severe hypoxia (F_I_O_2_: 0.112) in a single-blinded crossover design. Cognitive function and blood glucose were assessed before and after 0, 10, 30, and 60 min of the SIE. Heart rate (HR), peripheral oxygen saturation (SpO_2_), and ratings of perceived exertion (RPE, the Borg 6–20-point scale) during each SIE trial were recorded before and immediately after every five cycling bouts, and after 0, 10, 30, and 60 min of the SIE. Results: All the three SIE trials had a significantly faster overall reaction time in the Stroop test at 10 min after exercise as compared to that of the baseline value (*p* = 0.003, *ƞ*^2^ = 0.606), and returned to normal after 60 min. The congruent RT at 10 min after SIE was significantly shorter than that of the baseline (*p* < 0.05, *ƞ*^2^ = 0.633), while the incongruent RT at both 10 min and 30 min were significantly shorter than that measured at baseline (*p* < 0.05, *ƞ*^2^ = 0.633). No significant differences in terms of accuracy were found across the three trials at any time points (*p* = 0.446, *ƞ*^2^ = 0.415). Blood glucose was significantly reduced at 10 min and was sustained for at least 60 min after SIE when compared to pre-exercise in all trials (*p* < 0.05). Conclusions: Acute SIE improved cognitive function regardless of oxygen conditions, and the sustained improvement following SIE could last for at least 10–30 min and was unaffected by the altered blood glucose level.

## 1. Introduction

High-intensity interval training (HIIT) has been promoted over the last decade as an effective form of exercise training for improving cardiovascular and metabolic function and mental health in active young adults [1,2] and patients with coronary artery disease [3]. The benefits of the lower volume and time commitment of HIIT in overcoming the time-related barrier to physical activity adherence show clear superiority compared to traditional endurance exercise. As such, extreme short (<10 s) bouts of all-out sprint interval exercise (SIE), classified as one form of HIIT, seems to be an attractive option for achieving exercise-related health benefits [4].

Hypoxic training has been widely adopted as an effective strategy to enhance exercise performance and physical fitness in athletes for decades. More recently, this training strategy has shown its beneficial effects on cardiometabolic health and weight management in untrained individuals [5,6,7,8]. Compared to training at sea level, hypoxic training could further enhance blood oxygen transport capacity and aerobic endurance, as reported in previous studies [8,9,10]. However, the optimal dose of hypoxic severity for health-enhancing strategies is yet to be determined in view of the widely varying hypoxic exposure duration and severity used in the experimental protocols in the previous studies. The discrepancies in methodology make generalization challenging. Protocols that elicit beneficial effects on cardiorespiratory, metabolic, and immune health without pathology are more likely to arise from hypoxic exposure to a 9–16% fraction of inspired O_2_ [11]. Under such conditions, the exaggerated reduction in oxygen saturation increased ventilatory demand in response to hypoxia as a compensatory effect [12], with a more severe hypoxic level during a short period of exposure most often leading to beneficial cardiovascular effects [11]. This suggested that the severity of hypoxia appears to be the pivotal factor to determine this critical dependency.

A number of studies have demonstrated that acute exposure to hypoxia can negatively alter human cognitive function due to reduced oxygen partial pressure [13]. Cognitive deterioration may occur in acute hypoxic exposure at altitudes above 6000 m (FiO_2_: 0.097) [14]. Physical activity has previously been shown to enhance cognitive function [15,16,17]. It has been found that SIE improved selective attention [18], lexical learning [19], and executive function [20] in adults, and such beneficial effects were greater [19] and lasted longer [20] when compared to those resulting from continuous aerobic exercise. Due to the discrepancies in severity and duration of hypoxic exposure, however, results from randomized control trials and reviews investigating exercise combined with hypoxia showed both beneficial [21,22,23,24,25,26,27] and detrimental effects [28,29,30,31,32,33,34] on cognitive performance. Notably, studies on cerebral oxygenation have reported that central oxygenation between normoxia and hypoxic conditions were different. Near-infrared spectroscopy (NIRS) measurement revealed that during incremental exercise under hypoxia, stages in low to high intensity elicit a larger degree of cerebral deoxygenation compared with normoxia, which may limit cognitive activity and exercise performance [35]. Investigations that used electroencephalography and transcranial magnetic stimulation reported that as the severity of the hypoxia increased, cerebral oxygenation became more predominant in influencing cognitive activity and exercise performance [36]. Our previous studies demonstrated that high-intensity exercise under moderate hypoxia (FiO_2_: 0.154) [34] and moderate-intensity exercise under severe hypoxia (FiO_2_: 0.12) [25] had no detrimental effect on cognitive function. More information about the impact of different hypoxic severities on cognitive function is needed.

Acute SIE improved postprandial glucose metabolism in healthy subjects [37], an obese population [38,39], and type 2 diabetes patients [40] immediately or up to 24 h after exercise. Hypoxia is known to improve glucose effectiveness, and is suggested to facilitate further improvement in insulin sensitivity when combined with exercise [41]. Glucose is the major energy resource for the brain when processing cognition-related information. The brain is sensitive to disruptions in energy supply, whereas a modest increase in blood glucose concentration was associated with better cognitive performance [42]. Research has also shown that difficult cognitive tasks, such as those involving executive function pertaining to the frontal cortex, were more susceptible to glycemic alteration [43]. Available data indicate that acute hypoglycemia, or when the blood glucose concentration was below 3 mmol∙L^−1^, would impair cognition performance [44]. Therefore, glucose fluctuations induced by exercise in hypoxic conditions may have a negative influence on cognitive performance.

Given the above, this study used a single-blinded crossover design to evaluate the effects of SIE under normoxia, moderate hypoxia (F_I_O_2_: 0.154, simulating an altitude of 2500 m), and severe hypoxia (F_I_O_2_: 0.112, simulating an altitude corresponding to 5000 m) on cognitive function. We particularly focused on whether the cognitive-improvement effects of SIE were impaired while performing under hypoxia as the severity of hypoxia increased, and whether blood glucose was associated with cognitive performance during exercise under hypoxia. Given that brain function and tissue integrity are dependent on a continuous and sufficient oxygen supply, we hypothesized that cognitive function would be impaired during exercise under hypoxia as the severity of hypoxia increased.

## 2. Materials and Methods

### 2.1. Participants

The study was approved by the Research Ethics Panel of the University of Macau, and all experimental procedures were in accordance with the declaration of Helsinki. Advertisements including research purposes and inclusion criteria were posted on the e-bulletin board of the university to recruit recreational active males. The inclusion criteria were: (1) a maximal oxygen uptake ($\dot{V}$O_2max_) level of over 40 mL∙kg^−1^∙min^−1^ but lower than 55 mL∙kg^−1^∙min^−1^; (2) right-hand dominant; (3) nonhighland resident (above 1000 m); (4) without prior experience in hypoxic training; (5) free of any known neurological, cardiovascular, and pulmonary disorders; and (6) free from color blindness or abnormal vision. Smokers or those taking medication or having any physical barriers to performing SIE under hypoxia were excluded. Volunteers who were interested in this study were required to perform an incremental ramp test in the kinesiology lab to determine their $\dot{V}$O_2max_ and eligibility.

**Figure 1:**
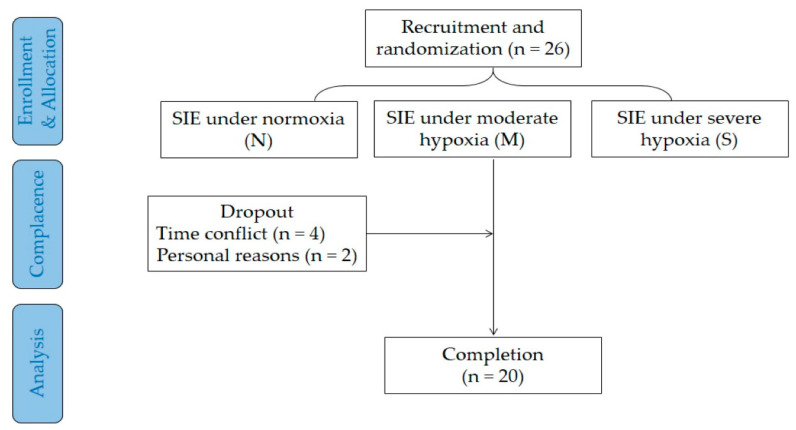
Flow diagram of the study.

An a priori power analysis was conducted using G*Power Version 3.1 to estimate the sample size. When the effect size was set at medium (*f* = 0.25), 21 participants would be needed to detect a significant difference in a two-way repeated-measures ANOVA with a power of 80% and a significance level of 5%. Considering a 20% dropout rate, 26 recreational active males were invited to participate in this study. All participants were informed of the experimental procedures and potential risks, and provided a written consent prior to participation. This study was conducted from October 2019 to September 2020. A total of 20 recreational active males (age: 21.4 ± 2.0 y, stature: 175.9 ± 9.1 cm, body mass: 68.7 ± 11.4 kg, body mass index (BMI): 22.1 ± 2.1 kg·m^−2^, maximal oxygen uptake ($\dot{V}$O_2max_): 42.9 ± 1.3 mL·kg^−1^·min^−1^) completed all experimental trials and required measurements (Figure 1).

### 2.2. Experimental Design

This study included a familiarization session and three experimental trials. During the familiarization session, participants were familiarized with the experimental procedures and practiced the SIE protocol and the Stroop test.

After the familiarization session, the participants accomplished three main trials at the same time on different days, namely an SIE trial under normoxia (N), an SIE trial under normobaric moderate hypoxia (M, F_I_O_2_: 0.154, simulated at an altitude of 2500 m), and an SIE trial under severe hypoxia (S, F_I_O_2_: 0.112, simulating an altitude corresponding to 5000 m). The three trials were assigned in a random and counterbalanced order, and were interspersed by three to seven days of washout period.

On the day of trials, participants were required to report to the lab before 17:50 to perform a baseline blood glucose test (using glucose and lactate analyzer, Biosen C-Line, EKF diagnostics, Barleben, Germany) and a Stroop test after a 10 min rest. Then, a standard dinner was provided to them at 18:00. A modified gas-mixing system (Everest Summit II Hypoxic Generator, New York, NY, USA) was used to generate normoxic or hypoxic gases, and the normoxic or hypoxic gas mixtures were delivered to participants through a breathing mask and tubes. At 19:30, participants were fitted with the breathing mask connected to the gas-mixing system, and they rested on the seat for 25 min. Then, a pre-exercise test of blood glucose and the Stroop test were performed. The SIE session (5 min warmup, 7 min exercise, and 3 min cooldown) began at 20:00 under the conditions of normobaric normoxia (i.e., the N trial) or normobaric hypoxia (i.e., the M and S trials). Stroop tests were carried out subsequent to the measurement of blood glucose levels at 10, 30, and 60 min after SIE. The breathing mask was taken off at 20:30.

Participants were unaware of the normoxic or hypoxic condition of the experimental trials, and they were required to guess the oxygen condition of the SIE trial by answering the question “Under what condition do you think you were exercising, normoxia, moderate hypoxia or severe hypoxia (i.e., sea level, 2500 m or 5000 m)?” However, no confirmation was given to the participants in order to avoid a potential influence on the subsequent trials.

### 2.3. Sprint Interval Exercise

The 7 min SIE trial consisted of 20 repetitions for 6 s of high-intensity cycling bouts interspersed with 15 s of passive recovery. Participants pedaled maximally against a load equivalent to 7.5% of their body mass during the 6 s work durations, and underwent passive recovery during the 15 s rest periods on a cycle ergometer (Monark 839E, Vansbro, Sweden). Heart rate (HR) and peripheral oxygen saturation (SpO_2_) during each SIE trial were recorded continuously by a pulse oximeter (Radical-7 Pulse CO-Oximeter, Masimo, Irvine, CA, USA), while ratings of perceived exertion (RPE, the Borg 6–20-point scale) were recorded before and immediately after every five cycling bouts, as well as 10 min, 30 min, and 60 min after the SIE. Participants were introduced to the scale and given standardized instructions on how to rate their overall sensations of effort (including the feeling of peripheral working muscles and joints, central cardiovascular and respiratory systems, and the central nervous system [45]) before each exercise session. Peak power, average power, and the fatigue index were calculated using Monark Anaerobic Test software (Sports Medicine Industries, Inc., St. Cloud, MN, USA). The fatigue index was analyzed to determine the level of fatigue during the anaerobic exercise [46]. A higher fatigue index indicated a lower ability to maintain anaerobic performance over a series of sprints. The exercise HR was estimated using the average HR during the entire exercise session. The HR_max_ was determined as the highest value attained when the $\dot{V}$O_2max_ test was performed during the familiarization session. The percentage of HR_max_ was estimated using the ratio between exercise HR and HR_max_.

### 2.4. Cognitive Test

**Figure 2:**
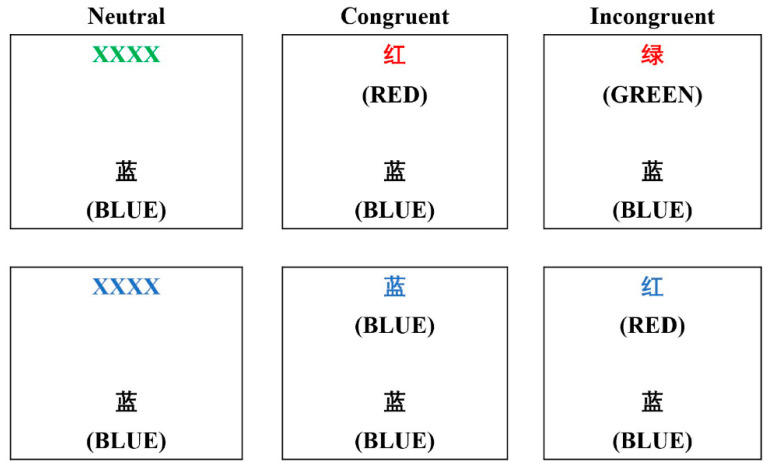
Instances of single trials for the neutral, congruent, and incongruent conditions of the color–word matching Stroop task.

For the cognitive task, the color–word matching Stroop test [47] was adopted to reflect prefrontal cortex function. A laptop with a pre-established E-Prime program was used to administrate the Stroop test. For each trial, two lines of letters were displayed on the laptop screen, and participants were instructed to determine whether the color of the letters on the top line matched the name of the color displayed on the bottom line (Figure 2), and to input their response by pressing “F” or “J” buttons to provide a “yes” or “no” choice with their index fingers. Each experimental session consisted of 30 trials, including 10 neutral trials, 10 congruent trials, and 10 incongruent trials, which appeared in a random order. For the neutral trial, the top line contained a group of Xs (XXXX) printed in red, green, blue, or yellow, and the bottom line contained the word “RED”, “GREEN”, “BLUE,” or “YELLOW” printed in black. For the congruent trial, the top line contained the word “RED”, “GREEN”, “BLUE,” or “YELLOW” printed in a congruent color. For the incongruent trial, the word “RED”, “GREEN”, “BLUE,” or “YELLOW” on the top line was printed in an incongruent color. All the word stimuli were presented in Chinese. To achieve sequential visual attention, the upper line was displayed 100 ms before the lower line (Schroeter et al., 2002). The rate of correct answers assigned to “yes” or “no” was 50% each. The stimulus disappeared from the screen when a response was given, or remained on the screen for 2 s. The correct answer rate (ACC) and reaction time (RT) for each type of trial (i.e., neutral, congruent, and incongruent trials) were calculated to measure the prefrontal cortex function.

### 2.5. Blood Glucose Measures

Arterialized venous blood samples from capillary finger-prick samples were collected before meals (baseline) and at 90 min (just before exercise), 120 min (10 min after exercise), 135 (30 min after exercise), and 150 min (60 min after exercise) after meals. Duplicate measures were analyzed with a portable glucometer (ACCU-CHEK Active, Roche Diagnostics, Indianapolis, IN, USA) that met the accuracy standards outlined by ISO 15197. If the measurement error between the first two blood samples exceeded 1.0 mmol∙L^−1^, one or two additional samples were taken. The average measurement from the two blood samples with the least variance was documented as the glucose concentration for the corresponding time point.

### 2.6. Physical Activity and Diet

In order to exclude the potential influence of diet and daily physical activity on outcome variables, six standard meals (i.e., 3 lunches and 3 dinners) were provided to each participant on the day of trials that included 1600–1620 kcal of total energy, with carbohydrate, protein, and fat accounting for approximately 60%, 10%, and 30%, respectively, of the total energy intake. Lunch and dinner were provided at 1:00 p.m. and 6:00 p.m., respectively, and the participants were required to finish in 15 min. Moreover, participants were instructed to refrain from coffee and alcohol, as well as strenuous exercise, for 48 h before the experiment days. Routine physical activities were assessed using pedometers (Yamax Digi-Walker SW-200, Tokyo, Japan) on one day before and on the day of the experiment.

### 2.7. Statistical Analyses

The PASW software (Release 22.0; IBM, Armonk, NY, USA) was used for statistical analyses. Prior to main statistical analyses, the Shapiro–Wilk test was conducted to confirm the normal distribution of the outcome variables. The data on cognitive function and psychological responses were analyzed using a two-way analysis of variance with two within-factors of condition (N, M, and S), and time (baseline; pre-exercise; and 10, 30, and 60 min after exercise). Significant interactions were further analyzed using a one-way repeated-measure test and/or paired test when necessary. The effect size of partial eta squared (*η*^2^) was calculated to determine the main and interaction effects. The effect size was considered small if *η*^2^ < 0.06 and large if *η*^2^ > 0.14 [48]. Cohen’s *d* values were used to access the effect sizes for the difference between the exercise trial and the control, and scores of 0.2, 0.5, and >0.8 were classified as small, moderate, and large, respectively [49]. All data are expressed as the mean ± standard deviation (SD), and *p* < 0.05 was taken as the level of statistical significance.

## 3. Results

### 3.1. Daily Activity and Baseline Values

No differences in routine physical activities were found among the three trials one day before (N vs. M vs. S = 8874 ± 3365 vs. 9427 ± 5112 vs. 8617 ± 4008 steps) and the experimental day (N vs. M vs. S = 7572 ± 3334 vs. 7536 ± 4231 vs. 7293 ± 2607 steps), despite fewer steps on the experimental day (*p* > 0.05).

**Figure 3:**
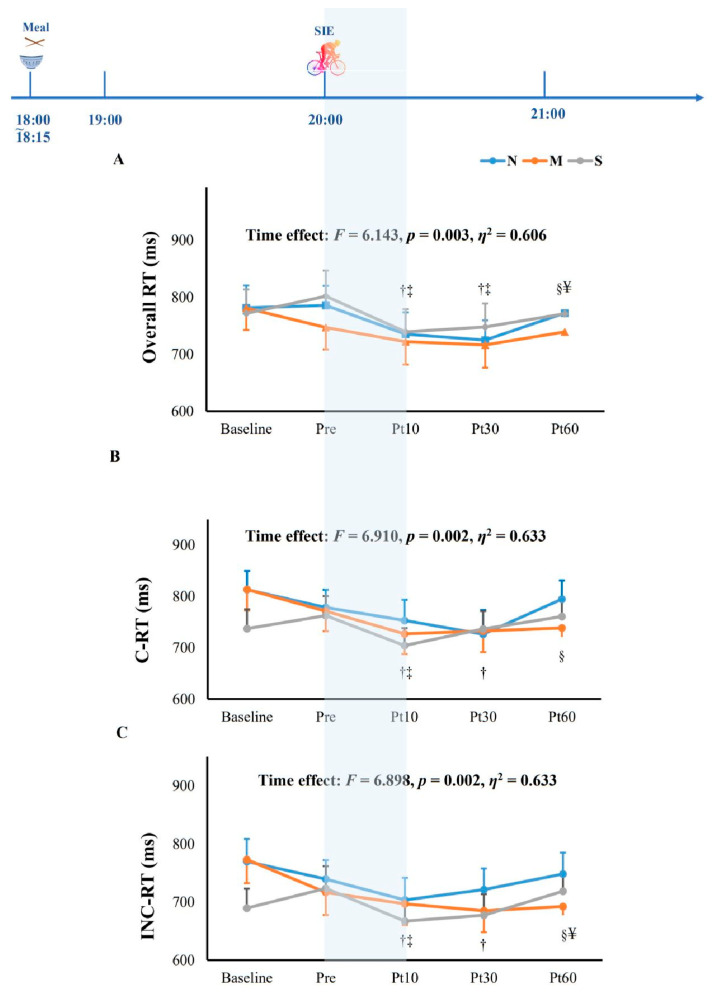
Overall (**A**), congruent (C-RT, **B**), and incongruent reaction time (INC-RT, **C**) in the Stroop test in responses to sprint interval exercise (SIE) under normoxia (20.9% O_2_) and hypoxia (15.4%O_2_ and 11.2%). The shaded part indicates oxygen condition. N: SIE in normoxia; M: SIE in moderate hypoxia; S: SIE in severe hypoxia; Pre: pre-exercise; Post 10: 10 min after exercise; Post 30: 30 min after exercise; Post 60: 60 min after exercise. † *p* < 0.05 vs. baseline; ‡ *p* < 0.05 vs. Pre; § *p* < 0.05 vs. Post 10; ¥ *p* < 0.05 vs. Post 30.

**Figure 4:**
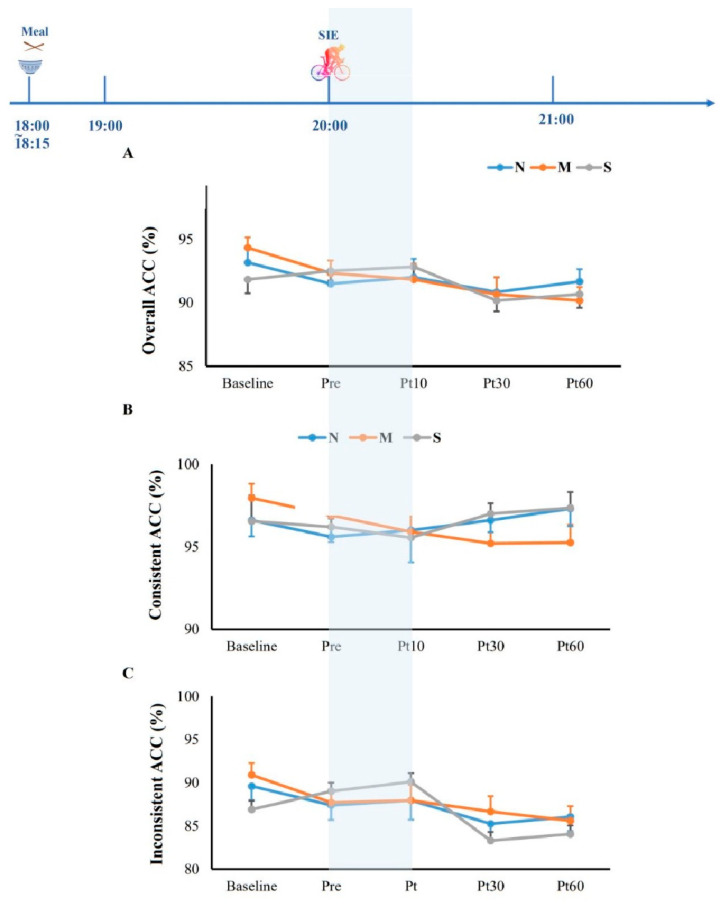
Overall (**A**), congruent (C-ACC, **B**), and incongruent accuracy (INC-ACC, **C**) in the Stroop test in responses to sprint interval exercise (SIE) under normoxia (20.9% O_2_) and hypoxia (15.4%O_2,_ and 11.2%). The shaded part indicates oxygen condition. N: SIE in normoxia; M: SIE in moderate hypoxia; S: SIE in severe hypoxia; Pre: pre-exercise; Post 10: 10 min after exercise; Post 30: 30 min after exercise; Post 60: 60 min after exercise.

The percentages of correct estimation of oxygen conditions were 30.0% in normoxia (N), 70.4% in moderate hypoxia (M), and 65% in severe hypoxia (S), indicating that the participants could not recognize the oxygen conditions (*p* = 0.06). Baseline measurements for cognitive function and blood glucose were taken at time points of baseline and pre-exercise, as shown in Table 1. As expected, the pre-exercise SpO_2_ was significantly lower in hypoxia compared to normoxia (*p* < 0.001), confirming the hypoxic conditions. Overall RT (*p* = 0.952) and ACC (*p* = 0.346) were not significantly different between baseline and pre-exercise (Figure 3 and Figure 4).

### 3.2. Exercise Performance and Physiological Responses

Peak power output was significantly lower in the S trial compared to that in the N trial (*p* < 0.01, Table 2). Mean power output was significantly higher in the M (6.1 ± 0.7 W∙kg^−1^) and S (5.9 ± 0.9 W∙kg^−1^) trials compared to that in N (6.4 ± 0.8 W∙kg^−1^, *p* < 0.01). There were no differences in the fatigue index, exercise HR, and percentage of HR_max_ among the three trials (*p* > 0.05).

The physiological responses (SpO_2_, HR, and RPE_20_) to SIE in normoxia and hypoxia are displayed in Table 1. There were significant interaction effects (*F* = 119.374, *p* < 0.001, *ƞ*^2^ = 0.992) on SpO_2_ among the three trials. Specifically, SpO_2_ was significantly lower in the M and S trials compared to the N trial at pre-exercise, and immediately and 10 min after SIE (*F* = 365.884, *p* <0.001, *ƞ*^2^ = 0.976). In the M and S trials, SpO_2_ was significantly lower at the time points of pre-exercise, immediately, and 10 min postexercise compared to that of the baseline (*F* = 446.551, *p* < 0.001, *ƞ*^2^ = 0.993). Significant interaction effects were also found in the HR levels (*F* = 4.682, *p* = 0.011, *ƞ*^2^ = 0.824); the HR before exercise was higher in the M and S trials compared to the N trial (*F* = 11.082, *p* = 0.001, *ƞ*^2^ = 0.552), and the HR levels measured in the later periods were lower than those measured in the preceding periods during the N, M, and S trials (*F* = 1613.675, *p* < 0.001, *ƞ*^2^ = 0.998). There were no differences in self-reported RPE between the three oxygen conditions (i.e., normoxia, 2500 m hypoxia, and 5000 m hypoxia; *p* = 0.993, *ƞ*^2^ < 0.001), suggesting that the three trails induced similar physical exertion at the same time point. However, participants perceived more effort immediately after exercise compared to that perceived at baseline and pre-exercise (*p* < 0.001, *ƞ*^2^ = 0.966).

### 3.3. Cognitive Function and Blood Glucose

**Figure 5:**
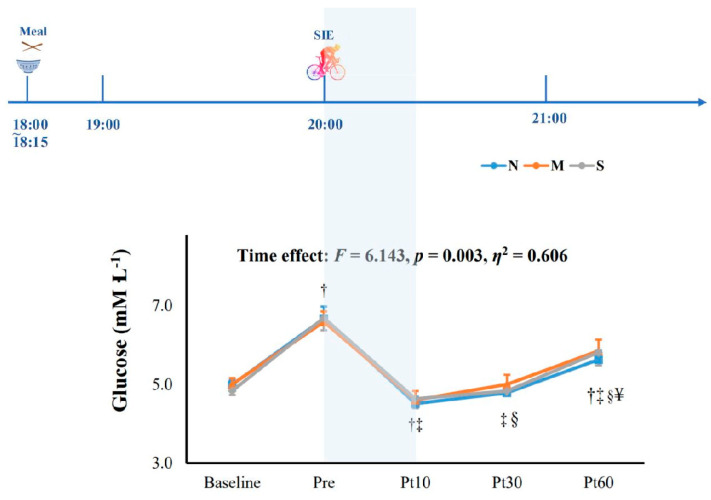
Glucose responses to sprint interval exercise (SIE) under normoxia (20.9% O_2_) and hypoxia (15.4%O_2,_ and 11.2%). The shaded part indicates oxygen condition. N: SIE in normoxia; M: SIE in moderate hypoxia; S: SIE in severe hypoxia; Pre: pre-exercise; Post 10: 10 min after exercise; Post 30: 30 min after exercise; Post 60: 60 min after exercise. † *p* < 0.05 vs. baseline; ‡ *p* < 0.05 vs. Pre; § *p* < 0.05 vs. Post 10; ¥ *p* < 0.05 vs. Post 30.

After SIE, the overall reaction time of the Stroop test was significantly reduced at 10 min after exercise as compared to that of the baseline value (*p* = 0.003, *ƞ*^2^ = 0.606), which was significantly higher at Post 60 when compared to Post 10 (Figure 3A). As for particular trials, the reaction time at 10 min after SIE was significantly shorter than that of the baseline in congruent trials (*p* < 0.05; Figure 3B), while the reaction times at both 10 min and 30 min after SIE were significantly shorter than that measured at baseline in incongruent trials (*p* < 0.05; Figure 3C). In terms of accuracy, no significant differences were found across the three trials at any time points (*p* = 0.446, *ƞ*^2^ = 0.415, Figure 4). Additionally, blood glucose was significantly reduced at 10 min and 30 min after SIE when compared to pre-exercise in all trials (*p* < 0.05, Figure 5).

## 4. Discussion

This study was the first trial to demonstrate the effects of SIE under normoxia and moderate and severe hypoxia on cognitive function and metabolic health in active males. Our result showed that acute SIE, regardless of oxygen conditions, facilitated cognitive function, and the lasting effect was sustained for at least 10–30 min. We also demonstrated that the RT improvement was unaffected by glucose alterations.

The main findings of this study were that SIE with or without hypoxia decreased RT, and this improvement did not sacrifice ACC in both conditions, indicating that this effect could improve cognitive function independently if it was normoxia, moderate hypoxia, or severe hypoxia. No detrimental effect was observed under exposure to a significant hypoxic-induced oxidative stress condition, which was strongly associated with greater reductions in cognitive function [32] by inducing low partial pressure of oxygen (PaO_2_) levels, indicated by the SpO_2_ decline from 98% to 89% and 78% in the moderate hypoxic SIE trial and severe hypoxic SIE trial, respectively, which may have been due to a compensation of the benefit of exercise-induced cerebral blood flow. It has been suggested that increased CBF and cerebral metabolism that maintained oxygen delivery to the brain during exercise are associated with cognitive improvement. Another possible reason for the stable cognitive performance may be attributed to the delivery of reserve oxygen. Under severe hypoxia, peak power and average power were significantly lower than N, with a small to medium Cohen’s d effect size (*d* = 0.37) of the fatigue index. A higher fatigue index in severe hypoxic SIE showed that sprinting under severe hypoxia made it more difficult to maintain the power than in other conditions, which induced a significant drop in the power output. It is plausible that the decrement in the performance was associated with the delivery of reserve oxygen to maintain homeostasis in the whole-body biological requirement and cerebral function, other than the exercising skeletal muscle, to prevent cognitive impairment.

In our study, overall RT and RT in congruent tasks improved following SIE regardless of oxygen conditions, and the effect was sustained for 10 min during the recovery phase and then returned to the baseline value; whereas the incongruent RT, a more sensitive type of task to detect the performance decrement based on the premise that it activated the neurobehavioral probe for the region associated with selective attention in a more challenging way [50], persisted for 30 min in both the normoxic and hypoxic SIE trials and returned to the baseline value after 60 min. In line with our results, SIE was previously found to improve cognitive performance as determined by a Stroop test, and the effect persisted for up to 30 min [20]; while another experimental study reported that intermittent exercise improved inhibitory control as assessed by a flanker test, and it was sustained for at least 60 min [51]. However, a negative effect was also found in a prolonged exercise that exposed the participants to an altitude of 5486 m, as assessed using visual perception, for up to 24 h [52]. Possible pathways by which acute hypoxic SIE may improve cognition included increasing cerebral blood flow [53] and enhancing the arousal level [20] and neurometabolism [54] in the brain to a positive level that led to an improvement in cognitive performance. Taken together, despite inconclusive results and an inapparent effect between oxygen conditions and different levels of hypoxia, the lasting effect of hypoxic SIE on cognitive function was significant in this study, regardless of oxygen conditions.

In our study, all trials showed a significant rise in blood glucose value at 90 min after meal ingestion and a significant drop after SIE and hypoxic SIE, and maintained a level lower than the pre-exercise value for at least 60 min. Our results were consistent with a recent study in healthy subjects that suggested that blood glucose remained stable under a hypoxic condition [55]. A recent review demonstrated that the mobilization of energy provisions augmented by hypoxic-induced physiological demand resulted in gluconeogenesis and glycogenolysis [56], and favored the energy balance in the competition of glucose demand. Others reported that exercise under hypoxia was expected to be efficient in glucose metabolism because it would further activate the exercise-induced glucose transport pathway, especially in SIE, which is characterized by its nature of high exercise intensity [57,58]. A possible mechanism is that lower oxygen availability would increase the higher proportion of glucose metabolism activated by sympathetic nervous system [59,60]. Our observations of cognitive performance during pre- and postprandial blood glucose excursion showed that cognitive performance was maintained as stable while postprandial glucose levels rose. In addition, the beneficial effects of SIE and the two levels of hypoxic SIE on cognitive performance were unaffected by the glucose drop at the postexercise time point compared to the preprandial glucose baseline value. Overall, our study showed that normoxic SIE and two different levels of hypoxic SIE significantly improved cognitive function, accompanied and unaffected by the altered blood glucose level.

There were several limitations to this study. Firstly, it was technically difficult to measure Post 0 due to the nature of SIE, so that immediate response was lacking in this study. In addition, the main effect of hypoxia needs to be clarified by a seated nonexercising trial with participants exposed to hypoxia for the same duration of time of the experimental trials. Our strengths were that we observed cognitive performance during a prescribed physiological state at successive multiple time points. This study provided evidence that cognitive improvement remains stable at both normoxic SIE and two levels of hypoxia, and it is also unaffected during blood glucose excursions. We used prolonged time points to investigate the lasting effects on cognition and blood glucose, which helped to provide information on the responsiveness of cognitive function following hypoxic SIE over successive time points, and how glycemic changes influenced cognition.

## 5. Conclusions

Similar positive effects on cognitive function were found in normoxia, moderate hypoxia, and severe hypoxia following acute bouts of SIE. Cognitive improvements lasted for at least 10–30 min, with neither losing accuracy nor being affected by an altered blood glucose level. Further research is warranted to elucidate the effects of different levels of hypoxia on cognitive performance with multiple tasks and gather data on cerebral blood flow to provide insight into brain activity, as well as the role of blood glucose in relation to the cognitive performance in active young adults, to better clarify the underlying mechanisms for the interaction between cognition and glucose metabolism. This study has implications for the use of SIE and hypoxia as a time-saving and more effective strategy to enhance physiological health [8,9,10] without cognitive impairment.

## Figures and Tables

**Table 1:** Physiological responses before and after SIE in normoxia and hypoxia.

	N	M	S
SpO_2_ (%)			
Baseline	98 ± 1	98 ± 1	98 ± 1
Pre	98 ± 3	89 ± 3 *^,a^	78 ± 4 *^,^**^,a,b^
Post 0	96 ± 3	84 ± 5 *^,a,b^	74 ± 58 *^,^**^,a,b^
Post 10	97 ± 1	89 ± 4 *^,a^	79 ± 5 *^,^**^,a^
Post 30	97 ± 1	97 ± 2 ^b,c,d^	96 ± 3 ^b,c,d^
Post 60	97 ± 1	97 ± 2 ^b,c,d^	97 ± 2 ^b,c,d^
HR (bpm)			
Baseline	63 ± 7	62 ± 6	64 ± 5
Pre	65 ± 6	72 ± 8 *^,a^	79 ± 10 *^,^**^,a,b^
Post 0	167 ± 9 ^a,b^	168 ± 11 ^a,b^	168 ± 7 ^a,b^
Post 10	92 ± 11 ^a,b,c^	94 ± 16 ^a,b,c^	95 ± 12 ^a,b,c^
Post 30	86 ± 11 ^a,b,c,d^	86 ± 13 ^a,b,c,d^	87 ± 11 ^a,b,c,d^
Post 60	81 ± 10 ^a,b,c,d,e^	80 ± 11 ^a,b,c,d,e^	80 ±9 ^a,b,c,d,e^
RPE_20_			
Baseline	6 ± 0	6 ± 1	6 ± 0
Pre	7 ± 1	7 ± 1	7 ± 1
Post 0	17 ± 2 ^a,b^	18 ± 2 ^a,b^	17 ± 2 ^a,b^
Post 10	8 ± 2 ^c^	8 ± 2 ^c^	8 ± 2 ^c^
Post 30	7 ± 2 ^c^	7 ± 1 ^c^	7 ± 2 ^c^
Post 60	7 ± 1 ^c^	7 ± 1 ^c^	6 ± 1 ^c^

N: sprint interval exercise in normoxia; M: sprint interval exercise in moderate hypoxia; S: sprint interval exercise in severe hypoxia; Pre: pre-exercise; Post 0: immediately after exercise; Post 10: 10 min after exercise; Post 30: 30 min after exercise; Post 60: 60 min after exercise. * *p* <0.05 vs. N, ** *p* < 0.05 vs. S; ^a^ *p* < 0.05 vs. baseline; ^b^ *p* < 0.05 vs. Pre; ^c^ *p* < 0.05 vs. Post 0; ^d^ *p* < 0.05 vs. Post 10; ^e^ *p* < 0.05 vs. Post 30.

**Table 2:** Variables of SIE in normoxia, moderate hypoxia, and severe hypoxia.

	N	M	S
Peak power (W/kg)	11.6 ± 1.9	11.4 ± 1.8	9.9 ± 2.8 *^,^#
Average power (W/kg)	6.4 ± 0.8	6.1 ± 0.7 *	5.9 ± 0.9 *
Fatigue index (%)	45.0 ± 13.6	48.8 ± 14.9	51.0 ± 18.4
Exercise HR (bpm)	169 ± 9	169 ± 11	168 ± 9
%HR_max_ (%)	93.1 ± 4.3	93.2 ± 5.4	92.8 ± 4.6

N: sprint interval exercise in normoxia; M: sprint interval exercise in moderate hypoxia; S: sprint interval exercise in severe hypoxia. * *p* < 0.05 vs. N; # *p* < 0.05 vs. M.

## Data Availability

The data presented in this study are available upon request from the corresponding author. The data are not publicly available due to the wishes of some of the subjects.
